# 2898. PrEP Inequity Across Geographic, Racial and Sex Groups in a Nationwide US Veteran Cohort

**DOI:** 10.1093/ofid/ofad500.169

**Published:** 2023-11-27

**Authors:** Lewis S Musoke, Amy Shumaker, Brigid Wilson, Puja Van Epps

**Affiliations:** Veterans Affairs Northeast Ohio Healthcare System, Cleveland, OH; VA Northeast Ohio Healthcare System, Cleveland, Ohio; VA Northeast Ohio Healthcare System, Cleveland, Ohio; eterans Affairs Northeast Ohio Healthcare System, Case Western Reserve University School of Medicine, Cleveland, Ohio

## Abstract

**Background:**

Despite increasing uptake of Pre-exposure prophylaxis (PrEP) for HIV prevention, gains have been disparate across groups in non-Veteran populations. PrEP to need ratio (PnR), a measure of PrEP use relative to HIV incidence in a population, has been shown to be a useful indicator of PrEP equity. Veterans Health Administration (VHA) is the largest integrated health care system in the US and offers an opportunity to aim for PrEP equity in the Veteran population.

**Methods:**

VHA’s nationwide databases were queried to identify PrEP users and new HIV diagnoses at each facility from 2019-2022. Unique PrEP users were defined as persons with ≥1 fill of PrEP during the calendar year (CY), excluding those with a diagnosis for HIV or chronic hepatitis B prior to the first fill in CY. A new HIV diagnosis was defined as a first ever positive HIV confirmatory test or detectable HIV viral load prior to first ever antiretroviral fill in a CY. For each region and CY, data was stratified by birth sex, self-reported race and ethnicity and the PnR was calculated as the ratio of PrEP users in a group divided by the number of new diagnoses in that group.

**Results:**

In the VHA, overall PnR increased from 2019 to 2022 (6.8 to 20.2), with the highest in the West at 18.9 and lowest in the South at 8.9 in 2022 (Figures 1 & 2). While gains were made across both Black and White racial groups, with West (W) and Northeast (NE) erasing racial disparities, PrEP use remained inequitable among Black Veterans compared to White Veterans in the Midwest (MW) and South, with the most pronounced disparities observed in the South (Black 5.7 vs. White 12.0). Non-white Hispanics had comparable PnR to White Veterans across most regions. Nationwide, PnR in males (birth sex) was double that observed in females (birth sex), but this differed by region with MW and W erasing disparities by 2022 while gaps worsened in the South and (8.9 vs. 5.3) and NE by 2022 (16.4 vs. 4.4).

**Figure:**
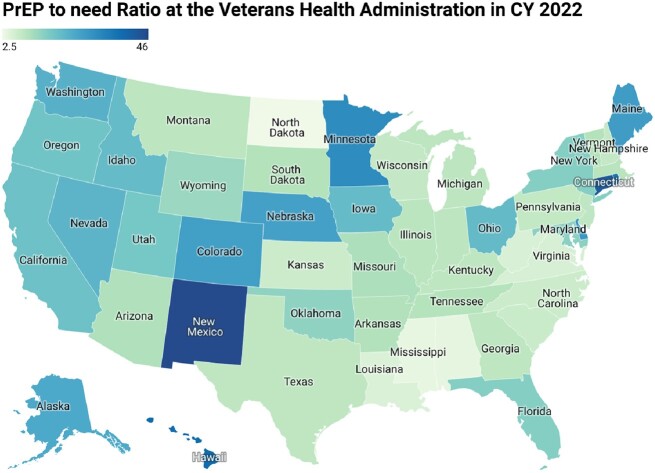

**Figure:**
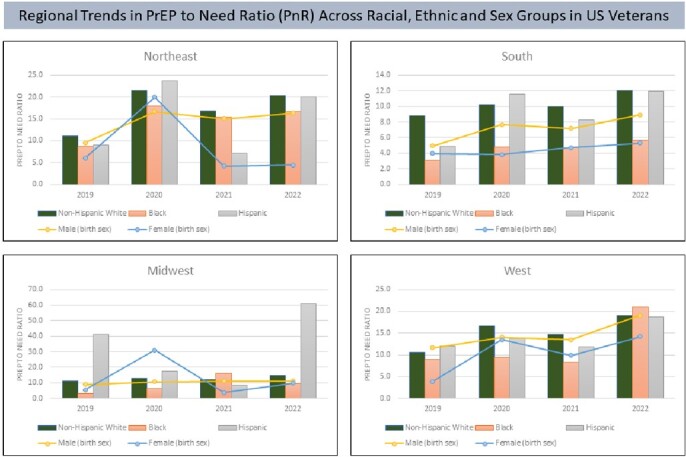

**Conclusion:**

Despite gains over the last four years, racial and sex-based disparities exist in PrEP use along geographic lines. VHA should continue to expand PrEP access across all groups, with a special focus on the South, particularly among Blacks and birth sex females. PnR can serve as a helpful metric for PrEP programs to identify areas of greatest need.

**Disclosures:**

**All Authors**: No reported disclosures

